# Retraction: Unveiling the Nexus: Influence of learning motivation on organizational performance and innovative climate of Chinese firms

**DOI:** 10.1371/journal.pone.0316037

**Published:** 2024-12-13

**Authors:** The PLOS ONE Editors

The *PLOS ONE* Editors retract this article [1, 2] because it was identified as one of a series of submissions for which we have concerns about the article’s adherence to *PLOS ONE*’s Ethical Publishing Practice polices, including but not restricted to concerns about the authorship, ethics approvals, integrity of the underlying data, and reliability of the published results. We regret that the issues were not identified prior to the article’s publication.

YuZ and QW did not agree with the retraction. CL, JL, YiZ, and SG either did not respond directly or could not be reached.
